# Model genotype–phenotype mappings and the algorithmic structure of evolution

**DOI:** 10.1098/rsif.2019.0332

**Published:** 2019-11-06

**Authors:** Daniel Nichol, Mark Robertson-Tessi, Alexander R. A. Anderson, Peter Jeavons

**Affiliations:** 1 Department of Computer Science, University of Oxford, Oxford, UK; 2 Department of Integrated Mathematical Oncology, H. Lee Moffitt Cancer Center and Research Institute, Tampa, FL, USA

**Keywords:** genotype–phenotype mapping, mathematical model, mathematical oncology, personalized medicine

## Abstract

Cancers are complex dynamic systems that undergo evolution and selection. Personalized medicine approaches in the clinic increasingly rely on predictions of tumour response to one or more therapies; these predictions are complicated by the inevitable evolution of the tumour. Despite enormous amounts of data on the mutational status of cancers and numerous therapies developed in recent decades to target these mutations, many of these treatments fail after a time due to the development of resistance in the tumour. The emergence of these resistant phenotypes is not easily predicted from genomic data, since the relationship between genotypes and phenotypes, termed the genotype–phenotype (GP) mapping, is neither injective nor functional. We present a review of models of this mapping within a generalized evolutionary framework that takes into account the relation between genotype, phenotype, environment and fitness. Different modelling approaches are described and compared, and many evolutionary results are shown to be conserved across studies despite using different underlying model systems. In addition, several areas for future work that remain understudied are identified, including plasticity and bet-hedging. The GP-mapping provides a pathway for understanding the potential routes of evolution taken by cancers, which will be necessary knowledge for improving personalized therapies.

## 1. Introduction

The failure of many treatments for cancers, infections and parasites can be attributed to the emergence of resistance. In some cases, these therapies are initially effective but fail later as drug-resistant disease emerges, while in others, these therapies fail from the onset. Ultimately, these patterns of failure are driven by Darwinian evolution. The selective pressures imposed by drug treatment result in the outgrowth of the most adapted subclones, causing the emergence of drug-resistant disease, and ultimately driving mortality. This process of evolution vastly outpaces our capacity to develop novel therapeutic agents. Indeed, the prevalence of drug-resistant bacteria such as methicillin-resistant *Staphylococcus aureus* (MRSA) and *Klebsiella pneumoniae* remains high and threatens to grow [1,2]. At the same time, our discovery of novel antimicrobial agents has declined significantly in recent decades [3], with some evidence of a recent but small uptick [4]. To bring a cancer drug to market can now cost more than $1 billion [5] but more often than not, when prescribed, these drugs can be expected to fail owing to drug resistance [6,7].

An alternative therapeutic approach is *evolutionary medicine*, wherein evolutionary theory is exploited to design treatment strategies wherein drugs are prescribed in combinations, sequences or metronomic/adaptive regimes in order to abrogate drug resistance. Recently, evolutionary approaches have been explored in the treatment of cancers [8–11], bacterial infections [12–14], viral infections [15,16] and malaria [17], with mixed results [18]. Ultimately, our ability to design effective evolutionarily informed therapies is predicated on *predicting* evolution [19], to pre-empt the emergence of drug resistance.

Evolutionary predictions are challenging due to the complex relationship between mutations and their phenotypic impact, the genotype–phenotype (GP) mapping. Genetic mutations can affect many aspects of organismal phenotype, in an environment-dependent manner, and with a dependence on the genetic background in which they occur. Further still, phenotypic changes can arise without genetic change, for example, through epigenetic modifications or through non-heritable mechanisms that generate phenotypic heterogeneity (see box 1, non-genetic heterogeneity). In the context of predicting the evolution of disease, deconvoluting the different drivers of phenotypic heterogeneity is critical, but extremely difficult, as properties of the GP-mapping can manifest unintuitively in experimental systems. In response, researchers have opted to study simplified models of the GP-mapping in order to generate hypotheses and drive experimental design. Here, we collate models of the GP-mapping under a common evolutionary framework, outline how results pertinent to evolutionary predictions are shared between models, and finally present a roadmap for further mathematical modelling of the GP-mapping as a tool for evolutionary medicine.

Box 1. **Non-genetic heterogeneity.**

Phenotypic heterogeneity can arise through heritable and non-heritable mechanisms. Mechanisms of phenotypic heterogeneity have been explored extensively in the study of ecology, and have recently been identified as potential drivers of drug resistance. Following ecological nomenclature, drivers of non-heritable phenotypic heterogeneity can be classified as *bet-hedging* or *phenotypic plasticity*. Bet-hedging describes the phenomenon wherein, for a fixed genotype and environment, multiple phenotypes can arise within the population stochastically, allowing the population to ‘hedge its bets’ against future environmental change or to diversify in order to maximize fitness in fluctuating environments (Seger [20] provides justification for this naming). An important clinical example of bet-hedging is that of *persister cells* that arise stochastically within isogenic populations of bacteria such as *E. coli* [21–23]. These cells, which constitute a small fraction of the population (less than 1% [22]), have reduced metabolism and shut down non-essential cellular functions. In this dormant state the persister cells are tolerant to the effects of a number of antimicrobials but can later proliferate to reconstitute a disease population.

Phenotypic plasticity describes the eco-evolutionary phenomenon wherein, for a given genotype, cellular or organismal phenotype is determined in an environment-dependent manner [24]. This determination might, for example, arise from developmental process or as a simple reactive change in phenotype when the environment changes. Phenotypic plasticity can be further classified as reversible or irreversible depending on whether further changes to the environment cause the phenotype to revert or change again. Phenotypic plasticity has also been observed to play a role in driving drug resistance; for example, cancer cells are known to upregulate the generation of efflux pumps in response to drug exposure [25].

Bet-hedging and phenotypic plasticity are separate drivers of phenotypic heterogeneity, although the two terms are sometimes used interchangeably, causing confusion. However, these phenomena do not cover the full spectrum of potential non-genetic drivers of phenotypic heterogeneity. First, bet-hedging and plasticity may co-occur. An example might be a population wherein phenotypes are determined stochastically (bet-hedging) in response to environmental shock (plasticity). Second, bet-hedging and phenotypic plasticity do not account for *partially* heritable phenotypes. A recent study identified persister-like phenotypes in human cells that were partially heritable, with offspring being more likely (but not certain) to take the persister phenotype dependent on the inherited cytoplasmic concentration of mitogen and p53 [26]. The development of theoretical and experimental models through which to explore partially heritable phenotypic heterogeneity, also called phenotypic ‘memory’, will be important in predicting evolution both for the treatment of disease and for ecologists more generally.

Mathematical modelling of the GP-mapping represents a large and growing field and as such a fully exhaustive review is intractable. We focus here on structural similarities between the most common models as a means to build intuition for the role of the GP-mapping in evolution, and in particular, in determining the success of evolutionary predictions. To this end, this review is structured as follows. First, we introduce the GP-mapping and present an algorithmic framework through which different models can be compared. Second, we introduce the earliest theoretical models of the GP-mapping; the fitness landscape and geometric model of Fisher. Third, we explore the use of secondary structure prediction in RNA as a model GP-mapping to explore the impact of neutral mutations on evolution. Fourth, we introduce models of the GP-mapping in which phenotypic heterogeneity can arise without corresponding genetic change. Finally, we conclude by discussing how similarities in the results derived from the theoretical models can serve as a guide for evolutionary theorists working to predict evolution in the context of disease management, as well as highlighting avenues for further theoretical studies of the GP-mapping. For brevity two important topics, fitness landscapes and the role of development, are discussed only briefly. Fitness landscapes have been reviewed comprehensively before, most notably by Orr [27] and De Visser & Krug [28]. Development represents a vast field in which the GP-mapping plays a critical role and has been reviewed previously [29–31].

### 1.1. The genotype–phenotype mapping

To predict how a population adapts to a given environmental change, it is necessary to understand how genetic alterations arise, how they manifest themselves as phenotypic change, and how viable the resulting phenotypes will be in the context of specific environments. Johannsen [32] was the first to explicitly recognize a distinction between the mechanisms of inheritance, that is the *genes*, and the physical characteristics, or *phenotype*, of an organism. Although a causal link between heritable ‘factors’ and organismal characteristics had been established by Mendel [33], Johannsen observed that this relationship is not a simple one-to-one mapping. The complexity of this relationship, later named the *GP-map* [34], was attributed to complex gene–gene interactions—epistasis [35] and dominance [33]. The identification of the physical mechanisms of inheritance, coupled with the later international effort to sequence the human genome in full, provided some insight into how genetic factors influence phenotype but failed to elucidate much of the process. The mechanisms of genetic transcription and translation have since been well categorized, as have, in part, the complex cascades of molecular interactions that form cell signalling pathways. Indeed, understanding of these pathways has formed the basis of the targeted therapy revolution in cancer therapy [36]. Nevertheless, a full mechanistic description of the GP-mapping remains elusive, owing in part to its highly complex and interconnected nature.

The GP-mapping is the subject of studies across multiple disciplines, with much of the work falling into three (not necessarily disjoint) categories—statistical analysis, mechanistic modelling and abstraction. Quantitative genetics and associated fields attempt to identify those genes associated with categorical phenotypes or to use statistical techniques to associate collections of genes, or specific loci of the DNA known as *quantitative trait loci* (QTLs), with measurable phenotypic variation (i.e. quantitative traits). These correlations provide insight into the GP-mapping as a ‘black box’ process, by associating the inputs (genotypes) with the outputs (phenotypes) and, from a clinical perspective, prove valuable in identifying genetic drivers for a number of diseases [37]. However, statistical correlations provide little insight into the mechanisms through which genetic alterations are manifested as phenotypic change.

**Figure 1.**
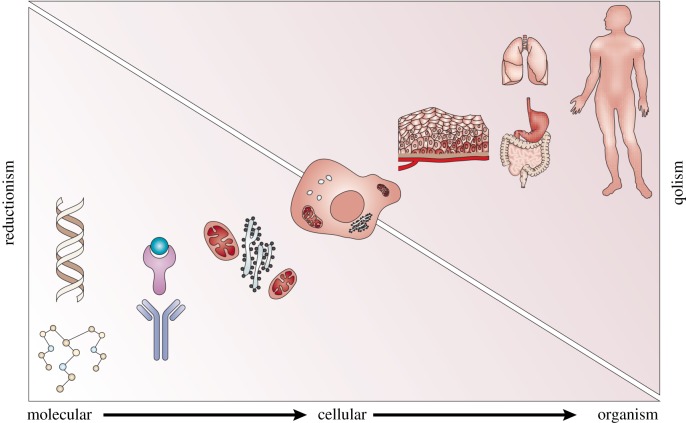
Cancer and the biological hierarchy. Genetic alterations induce modified intra-cellular signalling and drive the emergence of cancerous cellular phenotypes. The cells aggregate to form cancerous tissues (tumours) that eventually disseminate through the body. A reductionist approach suggests that this complex system can be understood by considering the basis (genetic) units. This approach fails owing to feedback mechanisms that bridge downward in the hierarchy. Evolution is an example of a such a mechanism as selection at the cellular, tissue or organ level determines which altered genotypes survive. Contrasting the reductionist approach is quantitative holism (qolism). Reproduced with permission from Anderson & Quaranta [38]. (Online version in colour.)

For much of the twentieth century, the biological mechanisms of the GP-mapping were probed through reductionist techniques. Under this approach, biological systems are organized into a hierarchy, with each layer comprising a basic biological entity, of which many are taken together to form a new entity at a higher level (figure 1 shows an example of this hierarchy in oncology). Thus, one can in principle study biology at any scale by first understanding the individual atoms and exploring how they interact to first form biological molecules such as DNA, then subcellular structures and, moving up the hierarchy once more, the living cell. Through this methodology, the GP-mapping is studied by direct construction from the basic building blocks of biology, the genes, the DNA or the constituent nucleotides. This approach has successfully explained the structure of DNA, the nature of inheritance and mutation and the process of transcription and translation via RNA which forms the first step in a complex process from which phenotypes emerge. Where reductionism can fail is in bridging between levels of the biological hierarchy, and in particular, in failing to account for feedback mechanisms that act across different scales. Implicit in the assumption of a hierarchy is a directionality in which smaller components combine and ‘feed-forward’ to build a whole. With regard to the GP-mapping, we know that gene expression is regulated through both positive and negative feedback mechanisms [39,40] by microenvironmental factors [41] or, in homeostatic systems, through inter-cellular signalling [42,43]. It follows that gene expression, and in turn cellular phenotype, is modulated by feedback mechanisms that bridge downwards across levels of the hierarchy.

In response to this failing, systems biology emerged as an alternative paradigm wherein the focus is shifted from identifying biological components towards understanding their complex, potentially nonlinear, interactions. By combining system-wide quantitative data with experimental and theoretical identification of intra-cellular interactions, systems biology builds mathematical and computational models that provide holistic biological predictions [44]. This paradigm has driven a revolution in modelling ecology and, since the early 2000s, is gaining traction as a tool to study molecular biology and biochemistry [45]. A notable success of this paradigm is the continuing improvement in cardiac modelling [46,47] and at present the systems approach is used to model cancer-associated cellular signalling pathways to identify potential targets for novel therapies [48–50].

Both the reductionist and systems approaches struggle to account for the overwhelming complexity of biological systems which cannot be overcome through the collection of ever more data and proves an intractable problem for explicit computational simulation. Instead, we must rely on abstraction to understand the GP-mapping, using tractable models of the underlying system. For example, the abstraction of nucleotides to a four-character alphabet (ATGC) reduces the DNA to a string and permits an algorithmic treatment that forms the basis of phylogenomics. In systems biology, intra-cellular molecular interactions are modelled as abstracted networks and analysed through graph-theoretic techniques [51,52]. An abstraction-driven approach to understanding GP-mapping proceeds by studying tractable analogues to biological systems as a means to gain insight into properties of more complex systems [53]. Biological systems are distilled to their base functional components (as opposed to the physical components of a reductionist perspective) and studied through the *algorithmic lens*. Understanding how these components interact serves as a conceptual tool in generating biological hypotheses and in guiding data collection, experimental design and further mathematical modelling. This paradigm has a rich history dating back to the work of Sewall Wright and Ronald Fisher who derived abstract models to explain aspects of evolution as early as the 1920s [54,55].

## 2. Evolution and the genotype–phenotype-mapping through the algorithmic lens

We begin by providing an algorithmic model of evolution through which models of the GP-mapping can be compared. Define a set of genotypes *G* as a set of strings over some alphabet of ‘alleles’ *Σ* (i.e. *G* ⊆ Σ*). Note here that the ‘alleles’ in *Σ* need not correspond to the biological definition of alleles but rather some basic heritable unit from which genotypes are built. Such a definition permits epigenetic inheritance. This abstraction allows for study of evolving systems at different scales. For example, DNA can be studied from the perspective of base pairs, codons or (biological) genes. We could model the genes from the molecular perspective by taking *Σ* = {*A*, *T*, *G*, *C*} and *N* ≈ 3 × 10^9^ or we could take a simpler Mendelian approach by setting *Σ* = {0, 1} to indicate the presence or absence of mutations of interest.

In this work, we focus on asexually reproducing cells and present models aiming to predict or prevent the evolution of drug resistance. We endow the set of genotypes *G* with a probabilistic mutation mapping

2.1 $$\mu \;:\;G\times G⟶[0,1]\;\text{satisfying }\forall g\sum_{h\in G}\mu (g,h)=1$$

which specifies the probability of a genotype *g* ∈ *G* mutating to a genotype *h* ∈ *G* during replication. Here, *μ* implicitly defines a mutation rate for each possible mutation. Where the set of possible genotypes is explicitly known and all mutations are equally likely, we can specify the full mutation relation by a single value which we call the mutation rate, often also represented by the symbol *μ*. As we have not specified a given length for the genotype strings, these mutations could be point mutations, deletions, insertions or larger structural mutations such as chromosomal duplications. Mutation rates are often altered by environmental factors such as radiation or the presence of mutagenic chemicals. For simplicity, we assume that this specific environmental dependency remains fixed in all modelling and hence need not be accounted for.

The combination G=(G,μ) is a *genotype space* [56]. Stadler [56] showed that, regardless of the choice of the mutation mapping, this definition of genotype space corresponds to a directed graph in which the vertices represent genotypes and edges link genotypes to their mutational neighbours.

In contrast with the structured nature of genotype spaces, it is difficult to give a general definition for a space of phenotypes, as the definition of phenotype varies among biological disciplines. We take the *phenotype space*, P, as the collection of potential traits of interest and phenotypes defined as the product of a number of characters that may be discrete or continuous.

Biologically, phenotypes are determined not solely from a genotype but are also modulated by environmental factors [57]. We represent an environment **e** = (*e*_1_, …, *e*_*k*_) (k∈N) as a list of values for environmental variables of interest from domains *E*_1_, …, *E*_*k*_ that may be continuous or discrete. As an example, in studying drug resistance, the *e*_*i*_ could be binary valued to indicate the presence or absence of a drug, or in a more complicated pharmacological model, be real values denoting the concentration of each drug. The space of possible environments is denoted $E\subseteq E_{1}\times \cdots \times E_{k}$.

Finally, we define a GP-mapping^1^ as a relation

2.2 $$R_{\mathrm{GP}}\subseteq (G\times E)\times P,$$

which associates a given genotype *g* to a set of possible phenotypes $P_{g}\subseteq P$ modulated by an environment **e**. We emphasize that the mapping is not a function, since multiple phenotypes can arise from a given genotype. It should be noted that this definition of the GP-mapping is a simplification of biological reality as there is no dependence on time, and phenotypes are modelled as being determined instantaneously at birth. This assumption removes the process of biological development from our algorithmic model of evolution. Of course, development is an important area of active research with a strong dependence on the GP-mapping [30], but well beyond the scope of this review (see Tomlin & Axelrod [58] and Oates *et al.* [59] for reviews of mathematical modelling of development, and Pigliucci [30] for a discussion of the GP-mapping in this context).

Evolutionary models require a set of (usually stochastic) rules governing the processes of death and replication/birth within a population. These rules are usually determined by *fitness* values that encode the survivability or fecundity of an individual with a specific phenotype in a given environment. The concept of fitness is an abstraction used to aid in understanding the process of adaptation through mutation and selection; however, there is no singular definition of fitness: multiple subtly different definitions exist across biological and mathematical disciplines [27]. Here, we define fitness via a function *f* that assigns to each pair of phenotype and environment a real value

2.3 f : P×E⟶R,

which encapsulates the factors of an individual’s viability, fecundity and other factors governing reproductive success into a single value. Where a GP-mapping is specified, we may extend this definition to genotypes as the expected fitness of an individual

2.4 $$f(g,e)=\sum_{\;p\in R_{\mathrm{GP}}(g,e)}P(p|R_{\mathrm{GP}},g,e)f(p,e).$$

For a fixed environment, this mapping defines a *fitness landscape*. The combination of a genotype space G, phenotype space P, environment *E*, GP-mapping R and fitness function *f* gives an evolutionary framework E=(G,P,E,R,f) through which to compare evolutionary models. Simulation within this framework proceeds according to rules governing the population dynamics. A number of population dynamics models exist and which is appropriate depends on the nature of the underlying biological question (box 2).

Box 2. **Population dynamics, population size and mutation rate.**

In many models of population dynamics, and in biological reality, the fate of a new genotype (and the associated phenotypes) within a population is not deterministic. Beneficial mutants can be lost through genetic drift or deleterious mutations can fix in small populations [60]. Under certain conditions, simplifying assumptions can be made. Specifically, the fate of a new mutant arising in a population, and the maintenance of genotypic heterogeneity within that population, will be dependent on the relationship between population size, *N*, mutation rate, *u*, and the *fitness benefit*, *s*, defined as the value such that if the resident population has fitness normalized to 1, the mutant has fitness 1 + *s*. For example, neutral mutations (those which do not affect fitness) fix with probability (1/*N*) [61] in fixed-size populations that evolve according to a Moran process. Beneficial mutations fix with a higher probability than this and deleterious ones with a lower probability. Provided that cells with genotypes of interest can be engineered, typical values for *N* and *s* can be estimated from cell culture experiments, however, estimations of the mutation rate parameter are more difficult owing to confounding factors such as the cell death rate [62].

If one assumes that all mutations are either beneficial or deleterious (selection is *strong*) and that *Nu* ≪ (1/log(*Ns*/2)), known as the strong selection weak mutation (SSWM) assumptions, then the population is almost always isogenic. This is because each new mutant will either *fix*, replacing the genotype shared by the whole population, or become extinct before another arises. Deleterious mutations fix with such low probability that this may be assumed never to happen. Thus, the population remains isogenic with a genotype that is periodically updated by a neutral or fitter mutational neighbour. Where the SSWM assumptions do not hold, for example, due to high mutation rate, large populations or neutral mutations, the population can be genotypically heterogeneous. This is because mutations, even when beneficial, are unable to fix or become extinct before new mutations arise. When considering the evolution of drug resistance, both of these modes of population dynamics have some relevance. For example, prior to treatment, the population size of disease cells is likely to be much larger than during treatment and thus it is more likely that the population is genotypically heterogeneous. This scenario is common in cancers where mutation rates are very high and the selective advantage of beneficial mutants has been observed to not be sufficiently strong to induce *clonal sweeps* and fix in the population [63–67]. In viruses, a similar pattern of large population sizes and high mutation rates is also observed, contributing to genotypically heterogeneous populations [68]. When drugs are administered, a strong selective pressure is imposed and a large number of cells die. This both lowers the population size and increases the strength of selection significantly, pushing the population dynamics towards the monomorphic SSWM type. For this reason, simulations of monomorphic/SSWM type population dynamics have seen use in predicting the *de novo* evolution of resistance during therapy [12]. A more thorough review of the relationship between the strength of selection, the rate of mutation and the dynamics of adaptation in asexually reproducing populations is provided by Sniegowski & Gerrish [69].

## 3. Mathematical models of evolution and the genotype–phenotype-mapping

We now present an overview of mathematical models of the GP-mapping viewed through the algorithmic lens. We begin by considering theoretical models developed by two of the earliest researchers to apply mathematics in studying evolution and population genetics: Ronald Fisher and Sewall Wright. These early theoretical models, Wright’s fitness landscape metaphor and Fisher’s geometric model, ignored the complex relationship between genotypes and phenotypes and instead explored evolution either from a purely genomic perspective, assuming a direct correspondence between genotype and fitness, or a purely phenotypic perspective, ignoring the genetic basis of changes to the phenotype. Despite these restrictive assumptions, these models yielded a number of relevant results that remain true in more complex models. A more thorough review of the historical impact of these models on evolutionary thinking was presented by Orr [70].

### 3.1. Fisher’s geometric model

The geometric model of Fisher [54] forgoes modelling the genotype and focuses on evolution at the phenotypic scale. The phenotype space is defined as a product of a number of continuous and independent traits,

3.1 $$P=R^{M}\text{for }M\in N,$$

where *M* can be considered a measure of organismal complexity. Genotypes *G* are taken to be equal to the phenotypes of P and the GP-mapping is taken to be the identity $R_{\mathrm{GP}}(p)=p$. This definition differs from our algorithmic definition of evolution above as genotypes are not discrete. Mutations are modelled as universally *pleiotropic* phenotype changes (a single mutation can affect all parts of the phenotype). Thus, mutations correspond to vectors $m\in R^{N}$ which change a phenotype p∈P (equivalently a genotype **p** ∈ *G*) according to

3.2 $$p\overset{m}{⟶}p+m.$$

**Figure 2.**
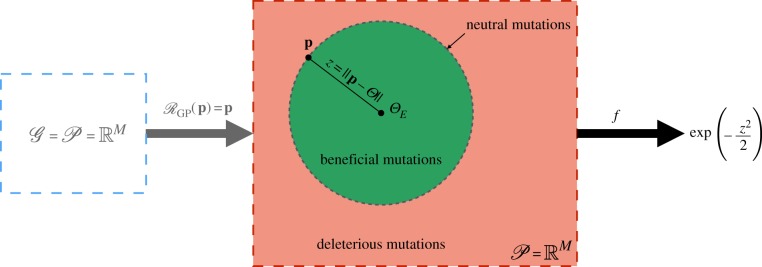
A graphical representation of Fisher’s geometric model. The genotype space G and phenotype space P are both $R^{M}$ and the GP-mapping is the identity. Mutations are vectors $m\in R^{M}$ that are added to a phenotype $p\in R^{M}$. The environment is determined by a globally optimal phenotype $\Theta_{E}\in R^{M}$ and fitness as a function of the distance (*z* = ‖**p** − *Θ*_*E*_‖) of a phenotype from this global optimum. Beneficial mutations are those for which **p** + **m** lies closer to *Θ*_*E*_ than **p** (inside the dashed circle). Deleterious mutations are those that generate a phenotype outside of this circle and neutral mutations those that generate a phenotype an equal distance from *Θ*_*E*_ (dashed circle). (Online version in colour.)

For a complete evolutionary model, these mutations must be assigned a likelihood. Fisher’s work omits any discussion of what this likelihood should be and instead focuses on the probability that a mutation of a *given magnitude*, *r* = |**m**|, confers a fitness advantage. The environment in Fisher’s model is defined as a single point in phenotype space, $\Theta_{E}\in P$, corresponding to the optimally adapted phenotype. Thus, E=P. The fitness, *f*, of an individual with a non-optimal phenotype **p** is defined in terms of a function, *w*, of the Euclidean distance (*z* = ‖**p** − *Θ*_*E*_‖) from the optimal phenotype f : p↦w(z). The function *w* determines how fitness decreases as phenotypes move away from the global fitness optimum. In Fisher’s original formulation, *w* is taken to be a univariate Gaussian function. A graphical representation of Fisher’s model is shown in figure 2.

Using this model, Fisher investigated the relationship between the magnitude of a phenotypic change and the likelihood of that change conferring a fitness advantage, determining that the probability that a mutation of magnitude *r* = |**m**| is beneficial is given by 1 − *Φ*(*x*), where *Φ* denotes the cumulative distribution function of a standard Gaussian distribution and $x=r\sqrt{\left(N/2z\right)}$ is a standardized mutation size. These results highlight several important features of adaptation on the phenotypic scale. Firstly, the probability that a mutation is beneficial approaches 0.5 from below as the magnitude approaches zero (provided the phenotype is not the optimal and that all mutations of a given magnitude are equally likely). From this result, Fisher concluded that the genetic basis for adaptation is the accumulation of a large number of very small phenotypic changes. However, as Kimura [71] demonstrated, while smaller mutations are more likely to be beneficial, they also confer a smaller fitness advantage when they are beneficial and thus are more likely to be stochastically lost through genetic drift (under certain regimes of population dynamics, box 2). Kimura derived the distribution of sizes among mutations substituted at each step of adaptation, concluding that it is mutations which induce *intermediate*, and not infinitesimal, changes in phenotype that are the most likely drivers of adaptation.

Orr [72,73] used Fisher’s model to determine the distribution of mutation sizes that occur successively during adaptation, as opposed to Kimura’s distributions of size at individual stages, finding that the distribution is approximately exponential and that adaptation in Fisher’s model is characterized by a relatively small number of large-magnitude mutations and many more small ones. The expected size of a mutation substituted at each step of an adaptive trajectory diminished by a constant proportion at each step, forming an approximately geometric sequence. Remarkably, simulations suggest this result to be robust to assumptions about the precise shape of the fitness function *w* [72,73].

Fisher’s model also predicts that the likelihood of a mutation being beneficial decreases as *M* increases, suggesting that adaptation proceeds at a slower rate for more complex organisms. Orr [74] described this phenomenon as the *cost of complexity* and estimated its extent, finding that the rate of adaptation declines at least as fast as M^−1^ under Fisher’s model. This cost was found to be reduced when the phenotype is *modular*, such that mutations only affect a subset of the characters comprising the full phenotype.

The geometric model has also been used to study other aspects of evolution including the evolutionary advantages of sex [75], development [76], compensatory mutations [77], mutation–selection–drift balance [78], mutation load [79] and hybridization [80].

### 3.2. Fitness landscapes

**Figure 3.**
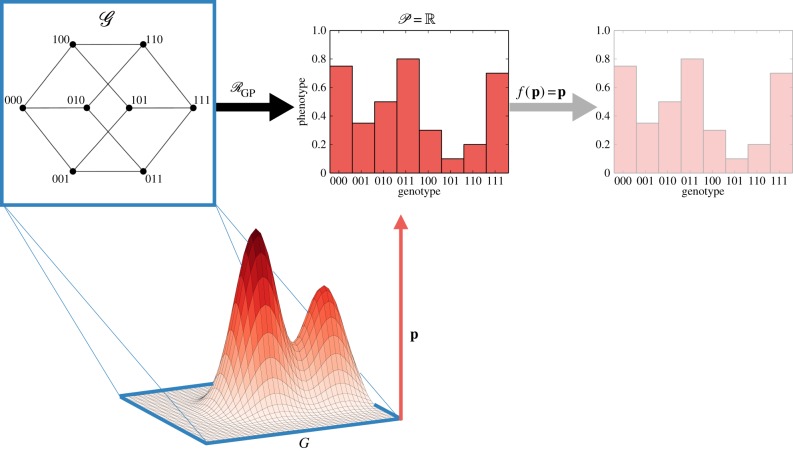
Fitness landscapes as an evolutionary system. The fitness landscape GP-mapping assumes that the phenotype is a single fitness value. In a schematic view of the landscape, the genotype space is projected into the *x*–*y* plane and fitness represented as the height above the plane. This representation emphasizes the dynamics of evolution as an ‘up-hill’ walk. (Online version in colour.)

The fitness (or adaptive) landscape metaphor was first introduced in the 1930s by Wright [55,81] as a model to account for *epistasis* wherein the fitness consequence of a mutation is modulated by the genetic background in which it occurs. For a static environment, all GP-mappings induce a fitness landscape over the genotype space according to equation (2.4) (box 2). However, we can consider the fitness landscape as the GP-mapping itself. A representation of this construction, together with the traditional schematic view of a fitness landscape introduced by Wright, is presented in figure 3. Under this construction, epistatic interactions are mathematically quantifiable and their effects on landscape topography can be measured. Coupled with empirically derived landscapes, this mathematical formulation provides insight into the evolutionary implications of the structure of the GP-mapping.

The landscape model is particularly useful in studying the accessibility, repeatability and predictability of evolution; three properties that are important in designing evolutionary therapies. Maynard Smith [82] introduced the concept of adaptive trajectories in discrete sequence spaces under the assumption that point mutations are sufficiently rare that one can assume no two occur simultaneously. These trajectories can be encoded as a sequence of point substitutions that each increase fitness. When the strong selection weak mutation criterion for population dynamics is satisfied (box 2), these trajectories are an accurate description of how evolution proceeds. A common visualization of a fitness landscape is to view the *x*–*y* plane as a genotype space G with a surface above that indicates fitness on the *z*-axis (shown in figure 3). Evolutionary trajectories are then viewed as ‘uphill’ walks on this surface . This metaphor has received some criticism [83] as in reality the genotype space is extremely highly dimensional, a property noted by Wright himself [55].

#### 3.2.1. Theoretical studies of fitness landscapes

**Figure 4.**
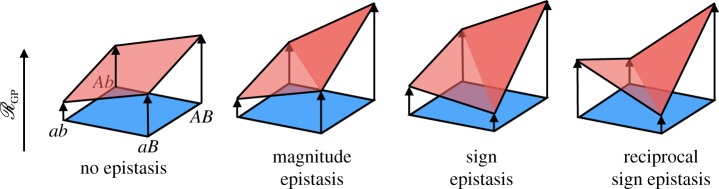
Forms of pairwise epistasis. The fitness (height above the plane) effects of different mutations, modelled as flipping a single bit of the genotype, can differ depending on the genetic background. These forms of epistasis can inhibit evolutionary trajectories between two genotypes. (Online version in colour.)

Epistasis is a key determinant of adaptation following environmental change and fitness landscapes offer a natural model in which to study the effects of epistatic interactions on landscape topography. The simplest form of epistasis is the pairwise interaction between two genetic loci, which may take different forms (figure 4). Of specific interest is *sign epistasis*, wherein the fitness effect of a point-wise mutation, *a* → *A*, changes sign dependent on the allele at a second locus (*B* or *b*). It has been shown that sign epistasis can severely restrict the number of accessible evolutionary trajectories between a low fitness genotype and a higher one [84,85]. *Reciprocal sign epistasis*, in which each of a pair of mutations *a* → *A* and *b* → *B* are individually deleterious but offer a fitness advantage together, can induce ‘fitness valleys’ between two genotypes, *ab* and *AB*, which cannot be crossed in many regimes of population dynamics. Poelwijk *et al.* [86] showed that the existence of a pair of mutations exhibiting reciprocal sign epistasis is a necessary condition for a landscape to have multiple optima of fitness. However, this study also demonstrated that there is no sufficient locally identifiable condition on gene interactions that guarantees a landscape is multi-peaked.

Epistasis can occur at higher orders than gene pairs, and in general the epistatic interactions among *n* genes (*n*th order epistasis) can be considered. Characterizing higher-order epistasis becomes increasingly difficult as for *L* loci there are $\left(\begin{array}{c} L\\ k \end{array}\right)$ subsets of size *k* that may interact. Weinreich *et al.* [87] introduced a mathematical formulation for higher-order epistasis. By analysing empirically derived landscapes using this technique, Weinreich identified higher-order epistasis in a number of empirically derived fitness landscapes for bacterial species. Taken together, these results indicate that epistatic interactions serve to restrict the accessible evolutionary trajectories in a fitness landscape. This restriction can render evolution (partially) predictable, potentially permitting the design of evolutionarily informed drug treatments that pre-empt, avoid, or reverse the emergence of drug resistance [13,14].

#### 3.2.2. Empirical fitness landscapes

During the past 20 years, novel experimental techniques have permitted empirical measurements of the GP-mapping [28]. Landscapes are measured by engineering strains of an organism with each possible combination of alleles at some genetic loci of interest. For example, in studying antibiotic resistance in Gram-negative bacteria, these loci may correspond to mutations in the genes coding for beta-lactamase. For *L* loci of interest, a total of 2^*L*^ strains must be engineered and then assayed in a drug-treated environment to determine resistance as a measure of fitness. This fitness measure is commonly determined as the minimum inhibitory concentration (MIC) of drug, or as the average growth rate in clinically relevant drug concentrations, although there is debate regarding what fitness metrics should be used to make effective evolutionary predictions.

De Visser & Krug [28] noted that by 2014 there had been fewer than 20 empirical studies to derive fitness landscapes and that of these, the maximum number of loci considered was *N* = 8. Drug-induced fitness landscapes have been derived for *Escherichia coli* [12,13,88–91], *Saccharomyces cerevisiae* [92], *Plasmodium falciparum* [93–95] and type 1 human immunodeficiency virus [96]. A meta-analysis shows that, on average, these landscapes show a substantial level of ruggedness which is suggestive of substantial epistatic, but not random, gene interactions [97]. For example, of the 15 drug landscapes derived by Mira *et al.* [13] all but one have multiple local optima of fitness, indicating that reciprocal sign epistasis is present between different loci of the TEM gene. This suggests that evolution is likely not always *repeatable*, but may be partially *predictable*, as often only a small number of evolutionary trajectories are accessible. Indeed, Weinreich *et al.* [88] derived an empirical landscape for *E. coli* exploring combinations of five mutations in the TEM gene under the antibiotic cefotaxime by using the MIC required to arrest growth as a proxy for fitness. Corroborating the theoretical result that sign epistasis restricts evolutionary trajectories, Weinreich finds that only 18 of 120 mutational trajectories from *g* = 00000 to *g* = 11111 are accessible.

Rather than focus on the full mapping from genotype to fitness, some studies have instead sought to derive empirical landscapes from a small part of the GP-mapping. For example, Aguilar-Rodríguez *et al.* [98] measured the binding affinity of a transcription factor to all possible short DNA sequences, generating over 1000 empirical landscapes exhibiting an intermediate degree of ruggedness and epistasis. It is not clear how these landscapes correspond to the fitness landscapes that arise at different biological scales, such as the drug-resistance landscapes described above.

De Visser & Krug [28] (also Orr [70]) demonstrated that accessible evolutionary trajectories in empirical landscapes are often short. This result is in contrast to the theoretical result showing that evolutionary trajectories in fitness landscapes can have length exponential in the number of loci [99]. This finding suggests that the GP-mapping induces a specific structure of fitness landscapes in which high fitness is accessible through relatively few mutations. Empirical datasets also indicate that beneficial mutations induce diminishing fitness benefit as the fitness of the genetic background increases [89,91,100]. Taken together, these fitness landscape findings corroborate the theoretical predictions of Fisher’s model [72,73], where the initial mutations in an evolutionary trajectory are, on average, expected to induce the largest increases in fitness. This result was further corroborated by *in vitro* experimental evolution of two bacteriophage viruses under the selective pressure of inhibitory temperatures [101]. From the perspective of early-stage cancer growth, these results may explain the so-called big bang growth dynamics, wherein genetically distinct subpopulations (subclones) grow together from the early stages [66]. These distinct subclones may genetically diverge later than the appearance of the tumourigenic phenotype, and thus differ by later-arising mutations conferring only small, or neutral, fitness effects that are insufficient for fixation of a single genetic clone. This pattern of diminishing fitness increases also favours the emergence of drug resistance, by allowing the rapid evolutionary escape of a pathogen from the toxic effects of drug therapy through only a small number of mutations.

#### 3.2.3. Limitations of fitness landscape models

Combinatorically complete empirical landscape studies require 2^*L*^ strains, and therefore only a small portion of the genotype space can be probed. This small subspace may miss important topographical features. For example, while one genotype may be separated from another by a fitness valley in the measured landscape, there may exist a trajectory of unmeasured mutations that connect the two. To fully understand landscape topography, we must improve data collection and better predict the extent of epistasis. This approach has been partially taken by Hinkley *et al.* [102] who used generalized kernel ridge regression (GRR) to estimate fitness landscapes from an incomplete dataset of fitnesses for different genetic strains. This work derives an approximation to the fitness landscapes of HIV-1 under 15 different drugs, for a genotype space totalling 200 genetic loci, using only 70 081 isolates. This landscape was later analysed by Kouyos *et al.* [103] who identified a high degree of ruggedness as well as large networks of genotypes (neutral spaces) over which fitness varies very little. Despite the successful empirical measurement of fitness landscapes for a number of transmissible diseases, no drug fitness landscape has been derived for mammalian or cancer cells.

### 3.3. Structure prediction models

Understanding how epistasis in fitness landscapes arises is not possible without consideration of the mechanisms through which phenotypes emerge from genotypes. Structure prediction models, wherein a prediction is made for the structure formed when a single-stranded sequence of nucleotides (or amino acids) ‘folds’ onto itself, have seen extensive study as directly computable mechanistic model GP-mappings [104].

#### 3.3.1. The RNA secondary structure model

The most common structure prediction model is the prediction of folded RNA secondary structures. This model is appealing for a number of reasons. Firstly, folded RNA constitutes an important part of the full mapping from genotype to phenotype. Secondly, RNA has been suggested as the first error-prone, self-replicating (i.e. evolving) molecule, and thus plays an important role in theories of abiogenesis [105]. Furthermore, RNA models have been used as a tractable, abstract model of evolution [106,107]. We present the pertinent results from abstract modelling of RNA folding as a GP-mapping. In the context of our evolutionary framework, the genotypes for the RNA secondary structure model are strings representing a single linear strand of RNA. Thus, the genotypes are given by *G* = {*A*, *U*, *G*, *C*}*. The most common choice of mutation relation is to only permit point mutations—a change of one nucleobase to any of the other three with equal likelihood, although in reality some substitutions are more likely than others [108].

The bases of an RNA molecule pair to form certain hydrogen bonds (A–U, G–C and less commonly, G–U) and thus an RNA strand will ‘fold’ onto itself to form a complex structure [109]. This folding can be conceptualized as having two stages. The base pairs first bond to form a planar shape known as a *secondary structure* and then distant parts of this structure come together to form a three-dimensional *tertiary structure*. It is this three-dimensional structure that determines the biological properties of the folded RNA; however, secondary structures are more easily computed and, given knowledge of the secondary structure, many of the biological properties of the full tertiary structure can be approximated. It is for this reason that secondary structures are taken as phenotypes in the RNA model.

An RNA secondary structure is defined as a list of pairs, $p=\{(i_{1},j_{1}),\ldots ,(i_{m},j_{m})\}$, (m∈N) of positions in the RNA strand *g*, each with *i* < *j* and satisfying that for any two (*i*, *j*), (*k*, *l*) ∈ *p*

1. *i* = *k* if and only if *j* = *l* (a given nucleotide can appear in at most one base pair)
2. k<j⇒i<k<l<j or *k* < *l* < *i* < *j*.

**Figure 5.**
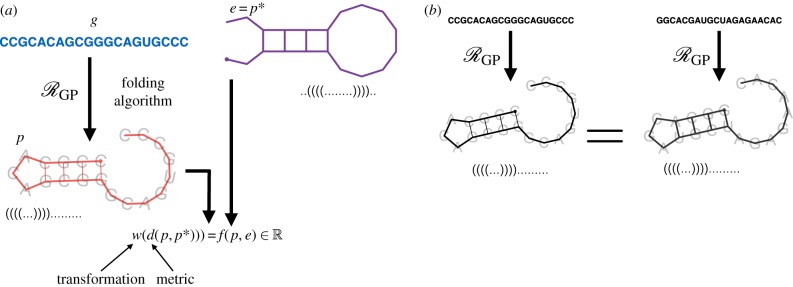
The RNA folding model. (*a*) RNA folding as an evolutionary system E. The GP-mapping takes a genotype *g* to a minimum free energy secondary structure *p* through an RNA folding algorithm (ViennaRNA [110]). The environment is specified as a target structure *e* = p* and fitness is determined as a transformation (*w*) of the distance (*d*) between a structure and this target. (*b*) Phenotypes/secondary structures are topologically defined, thus two RNA strands can fold into the same target structure. (Online version in colour.)

This second condition prevents the existence of *pseudoknots*, a structural feature of folded RNA that makes prediction considerably more difficult. In an evolutionary system, the phenotype space P is taken as the space of all such secondary structures. An example of this folding is shown in figure 5*a*.

RNA secondary structures can be represented in a number of ways including base pair lists as above, outer-planar graphs, tree structures, or in ‘dot-parenthesis’ notation as strings over the alphabet {(,.,)} where the symbols ‘(’, ‘.’ and ‘)’ represent base-pair openings, unpaired bases and base pair closings, respectively [111,112]. RNA secondary structures are topologically defined and do not depend on the specific underlying nucleotides of the RNA strand. Thus, different RNA strands can fold to form the same secondary structure (figure 5*b*). RNA secondary structures can be uniquely decomposed into combinations of *stacks*, which are double-helical runs of base pairs, and *loops*, which are sequences of unpaired nucleotides occurring between stacks. Secondary structures can be assigned a free energy value by considering the thermodynamic properties of the base pairs—loops of unpaired bases increase free energy while stacks lower it. Those secondary structures with lower free energy are most stable and thus the most likely to be formed when the RNA strand folds.

The simplest instance of a GP-mapping, $R_{\mathrm{GP}}$, using the RNA folding model is to assign to each RNA strand *g* ∈ *G* the *minimum free energy* (MFE) secondary structure p∈P. As the formation of a stack necessarily creates a loop, the energy trade-off between these two features induces a rugged energy landscape for secondary structure folds. Due to this rugged energy landscape, determining the MFE secondary structure, and thus mapping genotypes to phenotypes, is a non-trivial task. Indeed, it is not sufficient to simply maximize the number of base-pairings in the secondary structure (an example is provided by Wuchty *et al.* [113]). The free energy of a secondary structure is determined additively from the contributions of the loops within the structure and hence the MFE secondary structure can be determined through a dynamic programming algorithm [114,115]. This algorithm provides a tractable means to map genotypes to phenotypes within the RNA model and permits a statistical analysis of their relationship. Note that there exist other methods to predict folded RNA secondary structure that could also serve as a model GP-mapping [112]; however, a comprehensive overview is beyond the scope of this review.

The common approach to simulating evolution within the RNA model is to define a fitness function, *f*, on a secondary structure *p*, as determined by its distance from a given target structure that represents the environment (*e* = p*),

3.3 $$f(p,e)=w(d(p,p^{*})).$$

Here, *d* is a metric determining the similarity between two secondary structures, for example, the edit distance between the structures represented in tree form [109] or the Hamming distance between the structures represented in dot–parenthesis notation [111]. The function *w* is a monotonic transformation of this value. As RNA structures are often highly conserved between species, it has been suggested that slight deviations from the optimal structure can induce large changes in fitness. For this reason, a hyperbolic function is often chosen for *w* [116–118].

A schematic of the RNA secondary structure as interpreted as an evolutionary model is shown in figure 5. Here, we present some of the important results of the RNA model and interpret them in the context of drug resistance. A more comprehensive review of *in silico* studies of RNA folding is provided by Cowperthwaite & Meyers [104].

#### 3.3.2. Neutrality, robustness and evolvability

Perhaps the most fundamental finding of the RNA model is that the mapping from genotypes to phenotypes is extremely degenerate [119,120] with large neutral subspaces in the genotype space wherein every genotype corresponds to the same phenotype. The conclusion to be drawn from this finding is that a high degree of genetic heterogeneity need not necessarily correspond to the same degree of variation in phenotypes. This phenomenon is well understood in the study of cancer, where many mutations are observed to be neutral passengers [62].

Where the GP-mapping is highly degenerate, a number of questions arise regarding the likelihood and accessibility of different phenotypes. Exhaustive analysis of the space of short RNA sequences and their MFE secondary structures indicates that not all secondary structures are equally likely to be formed. Indeed, the majority of RNA strands fold into one of a small number of frequently occurring structures. For example, if a structure is defined as *frequent* when it corresponds to more sequences than the average structure, then for length 30 sequences comprising only G and C, 10.4% of MFE secondary structures are frequent but over 93% of sequences fold into them [121]. This degeneracy in the GP-mapping has profound implications for how evolution proceeds. In particular, (mutational) *robustness* and *evolvability*, are heavily dependent on this degeneracy. Robustness refers to the capacity of an organismal phenotype to remain unchanged when genetic mutation occurs and evolvability refers to the capacity of a population to generate phenotypic variability through genetic mutation. These two properties of the GP-mapping are at first glance inversely related, yet both are critical for the survival of a species. Consider an isogenic population with population genotype *g* of length *L* corresponding to a given MFE secondary structure *p*. There are 3*L* possible single-loci mutants of *g*. If *R* of these mutants are neutral (having MFE secondary structure *p*) and *S* are not, then *R* + *S* = 3*L*. Wagner [122] refers to *R* as the *genotype robustness* of *g* and *S* as the *genotype evolvability*. Clearly, these values are inversely related and there is an apparent trade-off; high robustness (large *R*) necessarily reduces the number of mutations, *S*, that induce phenotype change.

Neutral spaces in the genotype space permit a population of phenotypically identical individuals to overcome the robustness–evolvability trade-off through the generation of genetic heterogeneity. To see how this occurs we must consider how evolution occurs on a neutral subspace of *G*. If the strong selection weak mutation criterion is satisfied (box 2), then the population is necessarily isogenic and evolution proceeds as a random walk through the neutral space. In this case, the robustness–evolvability trade-off cannot be overcome. If instead, the population dynamics permit genetic heterogeneity, then the existence of neutral spaces increases both mutational robustness and evolvability. Consider a collection of genotypes *G*′ ⊂ *G*, wherein each genotype corresponds to the same MFE secondary structure, *p*, and any pair of genotypes is connected by a sequence of nucleotide substitutions that do not alter *p*. *G*′ is then a contiguous neutral subspace of *G* or a *neutral network*. Suppose that the phenotype *p* is optimal such that selection acts to preserve it (i.e. **e** = *p* = p*). From an initially isogenic population, say with genotype *g*, the accumulation of genetic mutations will cause the genotypes within the population to diversify and to resemble a ‘cloud’ in *G*′ known as a *quasi-species* [123–125]. When non-neutral mutations occur, the resulting individuals will be less fit than those of phenotype *p* and will be removed from the population by natural selection. Van Nimwegen *et al.* [126] demonstrated through graph-theoretic techniques and simulation that through this process the average robustness of an individual within the population, as measured by the expected number of neutral mutation neighbours ($\bar{R}$), increases as the population evolves. This is because the quasi-species does not spread randomly through the neutral network but instead becomes centred where there is a high density of neutral mutations. In short, robustness *evolves*.

A genotypically heterogeneous population will still contain individuals with genotypes that have low mutational robustness. These individuals lie at the boundary of the neutral space for *p* and the neutral space for different phenotypes. They will have higher sequence evolvability than other individuals and allow the population to generate phenotypic variation despite the majority of individuals exhibiting high robustness. Exhaustive computational analysis suggests that the neutral networks for frequent secondary structures can span the whole genotype space, permitting the mutation of *every* nucleotide (in some order) while maintaining phenotype [106,109]. Mathematical analysis from the theory of percolation can provide statistical constraints for when a neutral network will span the genotype space in this way [127]. These large neutral networks can border neutral networks for a large number of other phenotypes and thus, once the quasi-species spreads to contain genotypes from the whole neutral space for *p*, increase evolvability. It follows that neutral spaces for a given phenotype can increase both robustness and evolvability. This resolution of the apparent trade-off between robustness and evolvability was presented by Wagner [122].

The shape of neutral spaces also can also determine the time until a given phenotype arises in a population. Schaper & Louis [128] demonstrated that phenotypes with high frequency in the genotype space can arise and fix in a population even where rarer, higher fitness phenotypes are accessible. This phenomenon, which they term ‘arrival of the frequent’, suggests that the dominant phenotypes may arise as a result of the structure of the GP-mapping, even before the action of natural selection. This hypothesis is supported by the work of Dingle *et al.* [129] who showed that uniformly sampling from the genotype space of length 126 RNAs yields phenotypes with distributions of properties such as number of stems or mutational robustness closely matching those observed in a database of natural non-coding RNA.

Wagner [130] noted that results such as these are not solely limited to RNA secondary structures, but could apply in any instance where evolution occurs on genotypes drawn from a discrete sequence space. Comparing this perspective with our generalized evolutionary framework wherein the genotypes of *G* are *necessarily* discrete, we see that similar methods could be applied to *any* model GP-mappings. Indeed, recent studies have presented other biologically inspired GP-mapping models in which redundancy, phenotype bias and a correlation between robustness and evolvability all hold [131–133]. An empirically measured GP-mapping derived by measuring transcription factor binding site affinity also exhibits these properties [134,135], suggesting they may be a common feature of GP-mappings and that structure prediction models could serve as a useful tool for understanding evolution in this system. Ultimately, the reasons for choosing the RNA secondary structure model are purely pragmatic, to balance biological complexity with computational tractability.

#### 3.3.3. Evolutionary simulations with the RNA model

Consider now evolution towards some target optimal structure p* from another phenotype *p*. A number of *in silico* studies have used the RNA secondary structure model, coupled with the weak selection/strong mutation (box 2) population dynamics, to explore the effects of neutral networks on evolutionary dynamics [109,116,117,136]. The key finding of these studies is that the evolutionary dynamics follow a pattern of *punctuated equilibria*; long periods of phenotypic stability followed by rapid and large phenotypic change. This pattern occurs as neutral mutations accumulate and the quasi-species spreads to cover the neutral network. For some time, this spreading will only encounter genotypes corresponding to *p* or deleterious mutants of these genotypes. Thus, the phenotype will remain unchanged while genotypic heterogeneity increases. Eventually (if one exists), a fitter phenotype *p*′ bordering the neutral network for *p* will be found and a selective sweep will occur. The fitter *p*′ will come to dominate the population and genetic heterogeneity will decrease. The process then repeats until a local fitness optima *p* is found. The pattern of punctuated equilibria is common over evolutionary timescales [137] and has also been invoked to explain the dynamics of cancer evolution [138].

#### 3.3.4. Phenotype switching in RNA models

**Figure 6.**
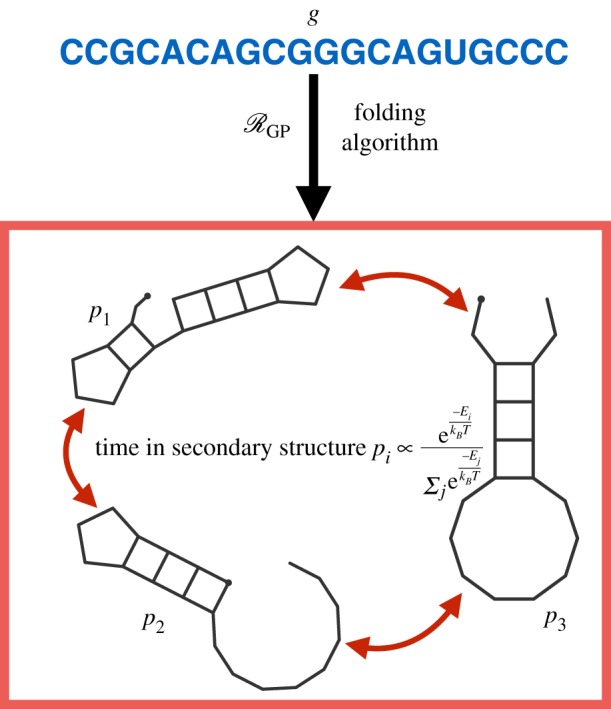
The stochastic RNA model. Thermal fluctuations cause RNA strands to spontaneously unfold and fold again into different secondary structures. In this model, the phenotype is not fixed but switches between secondary structures with free energy below some threshold. The time spent in each secondary structure is dependent on an environmental factor, the temperature *T*. This phenotypic switching is an example of bet-hedging in the GP-mapping. (Online version in colour.)

The structure of folded RNA strands are only metastable. An RNA strand will spontaneously unfold and refold into any of a number of low-free-energy secondary structures (figure 6). The time spent in a secondary structure, *p*_*i*_, with free energy, *E*_*i*_, satisfies,

3.4 $$\text{time in secondary structure}\;p_{i}\propto \frac{{\text{e}}^{\left(-E_{i}/k_{B}T\right)}}{\sum_{\;j}{\text{e}}^{\left(-E_{\;j}/k_{B}T\right)}},$$

where *k*_*B*_ ≈ 1.4 × 10^−23^ JK^−1^ is the Boltzmann constant, *T* is the temperature in kelvin and the sum is over all other secondary structures. Algorithms that calculate all secondary structures within some temperature range of the MFE structure have been derived [139]. Thus, it is possible to approximate the stochastic switching between a number of low energy secondary structures computationally. This stochastic switching, in the context of evolutionary modelling, is precisely phenotype switching or ecological *bet-hedging* [20,140], which we interpret as $R_{\mathrm{GP}}$ taking a non-functional form. It follows that the RNA secondary structure model has potential as a tool for studying the evolution of bet-hedging by considering a population of individuals wherein each has a secondary structure that can spontaneously switch. Furthermore, as the switching probabilities are dependent on the temperature *T*, this bet-hedging is environmentally modulated. This model was studied by Ancel & Fontana [116] who considered the effects of phenotypic switching on robustness, evolvability and modularity. They found that, under the selective pressure to evolve towards a target structure, bet-hedging incurs a fitness cost. The evolutionary consequences of stochastic switching in fluctuating environments, where bet-hedging has been demonstrated to offer a fitness advantage [141,142], have not been studied in the RNA model.

Many algorithms to determine MFE secondary structure ignore many of the physical and temporal dynamics of folding RNA. In reality, some parts of the structure fold into place before others which can cause an RNA strand to reliably fold into a secondary structure that is not the MFE [111]. The partially folded RNA strand can be considered to exist on a rugged energy landscape and to move ‘down-hill’ to more stable structures. This energy landscape is an analogue to the *epigenetic landscape* introduced by Waddington [143] as a metaphor to describe the role of development pathways in the *canalisation* (robustness to genetic or environmental variability) of phenotype. In Waddington’s work, the developmental pathways are viewed as inducing a landscape, and development as a ball ‘rolling’ down this landscape. Highly stable phenotypes occur due to high ‘ridges’ in the landscape that require large perturbations to knock the ‘ball’ over. The physical energy landscape of the kinetic folding model is in direct correspondence with the metaphorical model of Waddington, suggesting that the RNA model is an ideal candidate for studying the interplay between development and evolution. To our knowledge, few studies have taken this perspective, perhaps because the RNA model misses a key aspect of evolutionary developmental biology: that the developmental pathways (i.e. the shape of the landscape) can also evolve, whereas in the RNA model they are fixed by the laws of physics.

### 3.4. Network models

A key observation from quantitative genetics is that not all genes are equal in their potential to alter phenotype. Mutations occur with (approximately) equal probability throughout the genome, and yet the genetic drivers of phenotypic differences between, or within, species have been observed to accumulate in so-called hot spot areas of the genome [144]. This non-uniform distribution of non-neutral genetic variation can be attributed to the differing regulatory actions of some genes on others, which in turn drives epistasis and pleiotropy. This phenomenon, wherein genes regulate one another such that mutations at different genomic sites induce substantially different phenotypic effects (with respect to fitness), is poorly accounted for in the fitness landscape or secondary structure model.

A second biological phenomenon that is absent from these models stems from developmental biology. Consider the following: a critical process in embryonic development is the folding of a sheet of early cells to form a precursor to a central nervous system. A cluster of cells called the *Spemann organizer* has been identified as responsible for this process both in mammals and some invertebrates, for example tunicates. Those genes expressed within the mammalian Spemann organizer during development are present in the tunicate genome. What is remarkable is that these genes within the tunicate genome play no role in the development of the Spemann organizer [145,146] (although they are implicated in other aspects of development). From the perspective of our evolutionary model, there exist two species in which, in a restricted sense, the genotypes and phenotype are equal (or at least, similar), but the GP-mapping *itself* differs. Even if the genotype and phenotype are identical for two species, a different mapping means that the same mutation to *g* may manifest itself in distinct phenotypic differences—an important consideration if we are to predict evolution. This scenario of differing GP-mappings cannot be captured by the secondary structure model, since it is the laws of physics that determine the folded structure. Of course, in all biology it is physical laws that determine the GP-mapping, but where these physical interactions are intractably complex, we can consider different GP-mappings as an abstraction that accounts for unknown or unmeasurable differences between species. It is for this reason that network models encoding gene–gene or protein–protein interactions have arisen as a means to study GP-mappings in both evolutionary and developmental biology.

#### 3.4.1. Gene regulatory models

The gene regulatory network (GRN) model assumes the existence of *L* mutually regulatory genes and defines initial conditions for the activation/expression of these genes by

3.5 $$S(0)=(S_{1}(0),\ldots S_{L}(0))\in {\left\{-1,1\right\}}^{L}.$$

The cross- and auto-regulatory interactions of these genes are defined by a network *W* = [*w*_*ij*_]_*L*×*L*_ where,

3.6 $$w_{ij}=1\;\text{if gene}\;i\;\text{upregulates gene}\;j,$$

3.7 $$w_{ij}=-1\;\text{if gene}\;i\;\text{downregulates gene}\;j$$

and

3.8 $$w_{ij}=0\;\text{if there is no regulatory effect of gene}\;i\;\text{on}\;\;j.$$

The expression of the *L* genes at each time *t* is defined by values

3.9 $$S(t)=(S_{1}(t),\ldots \;S_{L}(t))\in {\left\{-1,1\right\}}^{L},$$

and subject to synchronous updates over a time step *τ* according to

3.10 $$S_{i}(t+\tau )=\sigma \left[\sum_{\;j=1}^{L}w_{ij}S_{\;j}(t)\right].$$

In the simplest, and most common, version of the GRN model, *σ* is the sign function and the phenotypes are taken as stable expression profiles,

3.11 $$p={\text{lim}}_{t\to \infty }S(t).$$

**Figure 7.**
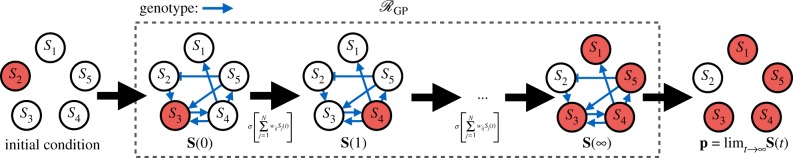
Schematic of the gene regulatory model for a GP-mapping. The genotype (blue arrows) defines the regulatory actions of genes on one another. The phenotype, *p*, is determined by the stable state for the network, under the update rule specified in equation (3.10). The initial condition of the network can be taken to be genetic, environmental, or arbitrary, depending on the specifics of each study. (Online version in colour.)

When **S**(*t*) does not converge in a finite number of update steps, the phenotype is assumed to be non-viable (having fitness equal to zero).^2^ The phenotype space in the GRN model is thus given by $P={\left\{-1,1\right\}}^{L}$, although more complex GRN models permit the genes to take real values and the function *σ* to take alternative forms. The convergence to the phenotype in equation (3.11) is assumed to occur instantaneously so that modelling can be simplified to not track different timescales (e.g. intra-cellular versus population-scale). A schematic of the GRN GP-mapping, along with example updates for the gene expression values as determined by *W*, is shown in figure 7.

**Figure 8.**
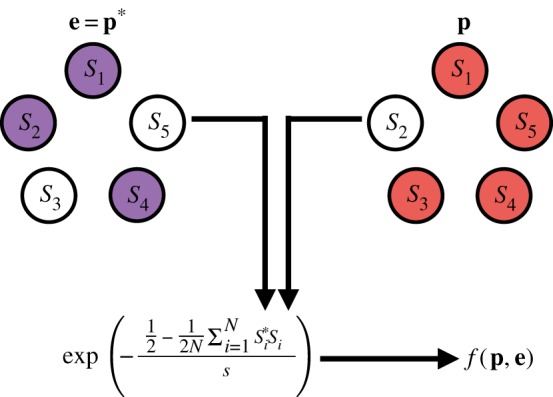
The fitness function for the GRN model. A metric value (in this case, Hamming distance) between a phenotype, **p** (red), and a target phenotype **e** = p* (environment, purple) is calculated and a transformation of this value is used to determine the fitness, *f*(**p**, **e**), of an individual with phenotype **p** in the environment **e**. (Online version in colour.)

GRNs have been used as a model for development with a focus on how changes to the underlying network, *W*, affect evolutionary phenomena such as evolvability or robustness. From the perspective of our generalized evolutionary systems, the genotypes, *G*, specify the *network* and not simply the *L* *genes* whose expression is being modelled. Thus $G={\left\{-1,0,1\right\}}^{L^{2}}$ and the mutation relation *μ* corresponds to changing an entry within the matrix *W* specifying the regulatory action of one gene on another. The environment, **e**, is determined by the specifics of different studies, but a common approach is to define a target phenotype, p*, that is globally optimal and measure the fitness as a function of the distance from this globally optimal phenotype (figure 8). For example, Wagner [148] used a Gaussian fitness function of the Hamming distance between the two phenotypes

3.12 $$\;f(p,e)=\exp\;\left(-\frac{\frac{1}{2}-\frac{1}{2N}\sum_{i=1}^{N}S_{i}^{*}S_{i}}{s}\right),$$

where *s* > 0 denotes the strength of selection. The initial conditions for gene expression differ between studies and can be genetically, environmentally, or arbitrarily defined depending on the purpose of the study.

Studies have explored how gene networks change through mutations that duplicate genes, delete genes or alter regulatory interactions [148]. Wagner [149] demonstrated that for GRNs evolving under stabilizing selection, evolution tended towards genotypes with few phenotypically different mutational neighbours, highlighting again that robustness to mutation is an evolvable property. Ciliberti *et al.* [150] extended this work by introducing a geometric description of the ‘meta-network’ of neutral spaces and found that mutational robustness and evolvability are negatively correlated for genotypes, but that for phenotypes with a fixed measure of robustness, the shape of the corresponding neutral space in *G* can increase evolvability. Payne *et al.* [151] studied GRNs using a random Boolean circuit model, also finding that robustness can evolve. These results mirror those arising in the RNA folding model. Crombach & Hogeweg [152] simulated the evolution of GRNs in fluctuating environments by periodically switching the environment between two target phenotypes, *p*_1_ and *p*_2_. This work found that evolution converged to genotypes on the ‘boundary’ between two neutral spaces in *G* corresponding to the two phenotypes, a result which was interpreted as the evolution of evolvability. More recently, studies have used the GRN model to explain the apparent paradox of the evolution of evolvability by drawing parallels with learning theory [153,154], highlighting the correspondence between the generalizability of a learning algorithm and the capacity for organisms to develop phenotypes well adapted to previously unseen environments.

As a conceptual tool to explain non-genetic heterogeneity of phenotypes, Huang [155] considered the stable expression profiles (i.e. phenotypes) induced by GRNs as attractors in a high-dimensional space [156,157]. In this model, gene expression profiles are assigned a ‘potential’ corresponding to the stability of the expression profile to stochastic molecular interactions. This assignment creates an *epigenetic landscape* (in the sense of Waddington [143]—arising not from single genes but the interaction of many). The local minima of potential in this landscape correspond to stable expression profiles (or equivalently phenotypes) and those gene expression profiles with higher potential will move down-hill through regulatory feedback mechanisms until a stable expression profile is found. Huang argues that non-genetic heterogeneity can then be explained by the existence of multiple accessible stable expression profiles, and that phenotypic switching is due to stochastic fluctuations causing jumps between stable states. These fluctuations can be either intra-cellular, for example, due to intrinsic thermal fluctuations of molecules, or extra-cellular and environmentally driven. Thus, Huang’s model of phenotypic heterogeneity can account for both bet-hedging and phenotypic plasticity, but no distinction is made between these two phenomena. Experimental evidence for this attractor states model was found by temporally monitoring the expression of over 2700 genes simultaneously during neutrophil differentiation following perturbation [158].

#### 3.4.2. Phenotypic plasticity and the neural network model

**Figure 9.**
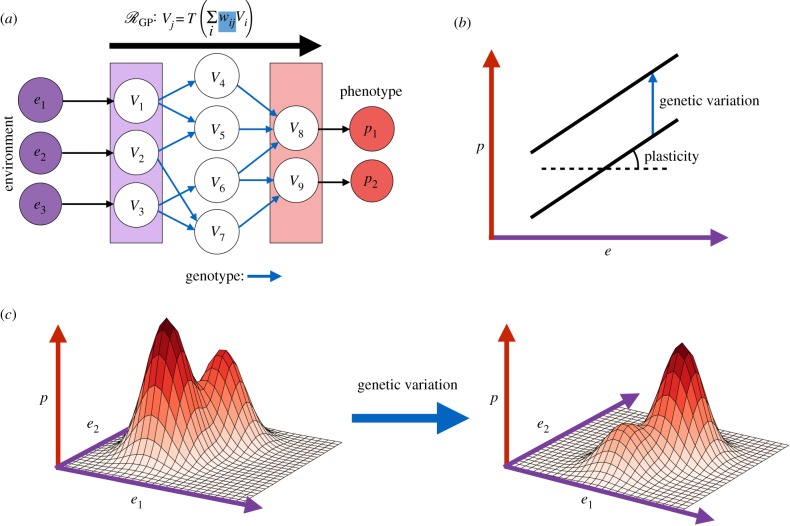
The neural network model and phenotypic plasticity. (*a*) Schematic of the (feed-forward) NN model. Environments, **e** = (*e*_1_, *e*_2_, *e*_3_), form the input (purple), the genotype determines the internal weights of the NN (blue) and the phenotype (red) is computed by evaluation of the NN. (*b*) The traditional (linear) representation of a reaction norm mapping an environment, *e*, to a phenotype *p*. Genetic variation results in changes to this relationship. (*c*) A higher dimensional and nonlinear reaction norm, these more complicated forms of the reaction norm can be represented by the NN model. (Online version in colour.)

Gerlee & Anderson [159] introduced an alternative network model for the GP-mapping in order to study the effects of phenotypic plasticity and spatial constraints in growing tumours [160,161]. In this model, genotypes are taken to define the internal wiring of a neural network that maps *r* real-valued environmental inputs ($E=R^{r}$) to *s* phenotypic traits ($P={\left[0,1\right]}^{s}$). The original formulation of this model was a *feed-forward* neural network, wherein the nodes are organized into layers and the internal wiring determined by g∈G only permits a node to influence those in the next layer (blue arrows, figure 9*a*). The phenotype is determined by setting the input nodes equal to some environmental state and evaluating the internal nodes in order of layer according to

3.13 $$V_{\;j}=T\;\left(\sum_{i}w_{ij}V_{i}\right),$$

where T : R→[0,1] is a thresholding function. In their work, Gerlee & Anderson [159] take $T(x)=(\frac{1}{1+e^{-2x}})$.

Gerlee & Anderson [159] embedded this model into a hybrid cellular automaton [162] to demonstrate the evolution of glycolytic or acid-resistant phenotypes in cancer in response to differing local concentrations of acid, glucose and oxygen, as well as spatial constraints. Harsh environments, for example, dense extra-cellular matrix (ECM) or low oxygen concentrations, drove the evolution of a glycolytic phenotype. This study also demonstrated that the network can induce different tumour morphologies, for example, a ‘fingered’ morphology evolved in low oxygen environments and a more compact morphology in environments with dense ECM. Gerlee & Anderson [163] studied this phenomenon in a more general framework to derive an approximate relationship between the parameters governing the dispersal of nutrients and the morphology of a growing colony. These studies clearly demonstrate that the impact of the GP-mapping stretches beyond the cellular phenotype and shapes the aggregate behaviour of a population. This *meta-*phenotypic behaviour can be much more complicated than simple morphology and could, for example, encapsulate developmental processes or self-organizing behaviour. It is from this perspective that the vital role of the GP-mapping in reconciling the modern evolutionary synthesis with developmental biology is most clear [30]. In a later study, Gerlee & Anderson [161] considered how the genetic and phenotypic heterogeneity of a population changed over the course of evolution, corroborating results derived from the RNA secondary structure model that suggest a high degree of degeneracy in the GP-mapping.

More recently, an extension of the neural network model to include *recurrent* networks was used to compare the efficacy of theoretical treatment strategies targeting single proteins, whole pathways or organismal phenotype [164]. In this model, the genotypes are unrestricted with respect to the influence one node may have on another. The GP-mapping, $R_{\mathrm{GP}}$, is then determined by solving a dynamical system defined by

3.14 $$\frac{\text{d}V_{\;j}}{\text{d}t}=T\;\left(\sum_{i}w_{ij}V_{i}(t)\right)-\lambda V_{\;j}(t),$$

where *λ* is a decay term for the artificial ‘proteins’ inside the model. Under this more complex model, the phenotype need not be stable but could exhibit oscillatory or even chaotic behaviour. Because the evaluation of a recurrent neural network is dependent on the initial values for all of the nodes, Gerlee *et al.* required that the initial values of non-environmentally determined nodes are zero. However, an alternative approach would be to permit nodes to retain their values (subject to decay) throughout the cell-cycle. This alternative modelling assumption could permit the study of epigenetic cell memory which has been demonstrated to confer an evolutionary advantage [165,166], and which has recently been observed in cancer [26].

At present, studies of the neural network model have been restricted to simulations of cancer progression or colony growth. An important parallel between the neural network model and previous biological modelling becomes clear when the GP-mapping is considered from our more general perspective. In 1985, Via & Lande [167] introduced the concept of the *reaction norm*—a genotype-dependent mapping from a single environmental parameter to a phenotypic value as a simple model of phenotypic plasticity (figure 9*b*). The work of Gerlee *et al.* [159] is a natural extension of this concept to higher-dimensional functions that can approximate any biologically occurring reaction norms (figure 9*c*), since recurrent neural networks can closely approximate any function on bounded input space. Furthermore, as neural networks can be trained without knowledge of the underlying mechanism and from limited datasets, the neural network model has the potential to *predict* non-genetic adaptation to previously unseen environments, provided a sufficiently large dataset can be derived.

#### 3.4.3. Stochastic network models and intra-cellular dynamics

A number of models have been developed which encode stochasticity explicitly into the GP-mapping. Charlebois *et al.* [168] introduced a model of phenotype switching derived from the stochastic relaxation from high to low gene expression and explored the relationship between relaxation time and the likelihood of resistance-conferring mutations arising before extinction. In a further study, Charlebois *et al.* [169] introduced a feed-forward transcriptional regulatory network model to demonstrate that the specific network architecture can extend the time that drug-insensitive cells maintain their phenotype, and thus the time window in which resistance-conferring mutations can arise.

**Figure 10.**
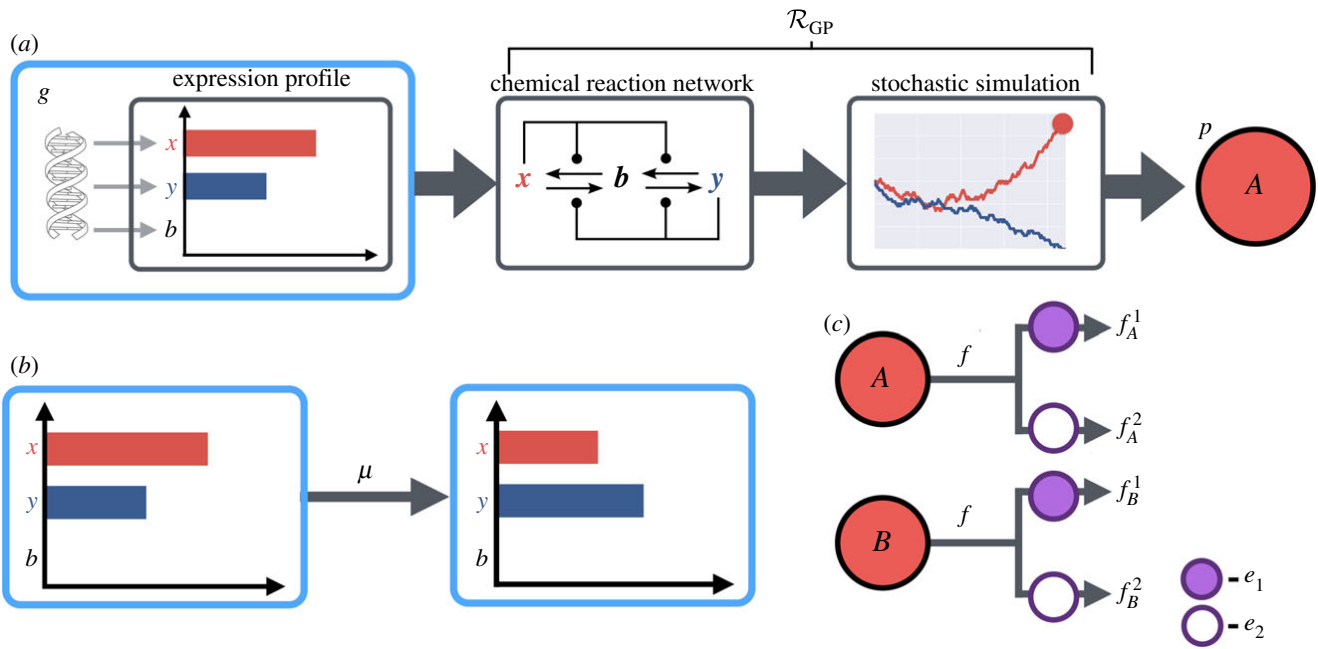
The CRN model for a GP-mapping. (*a*) A schematic for the CRN model. Genotypes encode initial values for expressed molecules, *x* and *y*. The GP-mapping is encoded as a bistable chemical reaction network that is stochastically simulated to determine the phenotype, *A* or *B*. (*b*) Mutations in the CRN model are alterations to the initial abundance of the intra-cellular molecules. (*c*) The environment is represented as a binary variable which could for example indicate the presence or absence of a drug. A fitness value is chosen for each combination of phenotype and environment. (Online version in colour.)

In our own previous work, we introduced a model GP-mapping wherein the phenotype is stochastically determined by the interactions of genotype-dependent intracellular molecules [170] (figure 10). In this model, genotypes are represented by the initial abundance of two molecules, *x* and *y* (i.e. *G* = {0 , …, *g*_max_}^2^), deterministically produced at birth. Mutations act to alter these initial values. We considered two possible phenotypes, P={A,B}, arising from the stochastic simulation of a bistable chemical reaction network (CRN) on the molecules *x* and *y*. Where the simulation ends in steady state consisting of all *x* (or all *y*) molecules, the phenotype was taken as *A* (or *B*, respectively). This stochastic simulation forms the GP-mapping, $R_{\mathrm{GP}}$. We explored an apparent paradox of bet-hedging: why is phenotypic heterogeneity maintained in fixed environments when it is necessarily deleterious (when compared with a single phenotype strategy)? We found that the structure of the GP-mapping itself can serve to slow the rate of evolution, maintaining phenotypic heterogeneity to serve as a survival mechanism in the event of rare catastrophic environmental change such as drug treatment. This result indicates that an understanding of the GP-mapping is critical not only in predicting how a population will evolve, but also in predicting the timescale of this evolutionary process.

## 4. The future of genotype–phenotype-mapping models

This survey of GP-mapping models has highlighted a number of important evolutionary phenomena, many of which are conserved between models. By considering these different models under the umbrella of a general evolutionary framework, we have been able to identify a number of similarities between models that would be missed by considering these models on an *ad hoc* basis. To conclude we present a summary of key properties of the GP-mapping arising from theoretical models (table 1) and highlight those aspects of the GP-mapping that have been overlooked in previous modelling studies. Finally, we provide a partial overview of evolutionary questions that could form the basis of future theoretical studies.

**Table 1.** Summary of abstract models for the genotype–phenotype mapping. For each of the models discussed in this paper, the key components of an evolutionary system are shown: the genotype space and mutation relation, phenotype space, GP-mapping, environment and fitness mapping.

model	Fisher’s geometric model	fitness landscapes	secondary structure model	gene regulatory networks	neural network model	CRN model
genotype space	$R^{M}$	*Σ*^*L*^	*Σ*^*L*^	$G={\left\{-1,0,1\right\}}^{L^{2}}$ (regulatory action of *L* genes)	$G=R^{k^{2}}$ (specifying a neural network)	$G={\left\{0,\ldots ,g_{\max}\right\}}^{2}$
mutation relation	**p** → **p** + **m**, m∈R	point mutations	point mutations	alterations to the internal connections	alterations to the internal weights	alterations to initial molecule numbers
phenotype space	$R^{M}$	R	$P={\left\{{\;}^{\prime }{{(}^{\prime },{\;}^{\prime }{,}^{\prime },{\;}^{\prime })}^{\prime }\right\}}^{*}$ (RNA secondary structures)	$P={\left\{-1,1\right\}}^{L}$ (stable expression profiles)	$P={\left[0,1\right]}^{s}$ (final values for *s* output nodes)	P={A,B}
GP mapping	identity	f : Σ→R	prediction of MFE secondary structure	lim_*t*→∞_**S**(*t*) where $S_{i}(t+\tau )=\sigma \left[\sum_{\;j=1}^{L}w_{ij}S_{\;j}(t)\right]$	$V_{\;j}=T(\sum_{i}w_{ij}V_{i})$ (evaluation)	stochastic simulation of a bistable CRN (GP-mapping not a function)
environment	target phenotype Θ∈P	fixed	target phenotype e∈P	target phenotype e∈P	$E=R^{r}$ (inputs)	discrete variable
fitness	$f\;:\;p\mapsto \exp\left(-\frac{∥p-\Theta {∥}^{2}}{2}\right)$	identity	*f*(**p**, **e**) = *w*(*d*(**p**, p*)) (*d* a metric, *w* a transformation)	$f(p,e)=\exp\;\left(-\frac{\frac{1}{2}-\frac{1}{2N}\sum_{i=1}^{N}S_{i}^{*}S_{i}}{s}\right)$	not specified/emergent	value for every phenotype/environment pair

### 4.1. Properties of the genotype–phenotype-mapping

#### 4.1.1. Neutrality and degeneracy

A key conserved property of models of the GP-mapping is a high degree of degeneracy in the relationship between genes and phenotypes. Many genotypes will map to a single phenotype, inducing large neutral subspaces of the genotype space, as evidenced by the RNA structure and network models. From the perspective of evolving drug resistance, this neutrality has important consequences. Neutrality can induce a two-stage pattern of evolution, wherein fitness first rapidly increases before following a pattern of punctuated equilibria, and may explain many aspects of disease progression [138]. The rapid increase in fitness associated with the early stages of evolution may explain why evolutionary escape from therapy is so prevalent, while the later stages of punctuated equilibria can explain why diseases that appear stable for many years may suddenly worsen—a common scenario in cancers and HIV infections. Understanding the relationship between neutral mutations, the associated genetic heterogeneity, and the potential for pre-existing or *de novo* evolution of drug resistance is of particular importance to understanding the progression of cancers and remains an active area of research [171,172]. It is clear from our survey that a focus on the GP-mapping could prove valuable in furthering our understanding of the impact of intra-tumoural heterogeneity and developing effective diagnostic and therapeutic techniques.

#### 4.1.2. Evolvability and robustness

A major contribution of theoretical modelling of GP-mappings is the resolution of the apparent paradox between robustness and evolvability. Neutral mutations can induce genetically heterogeneous but phenotypically homogeneous populations in which both robustness and evolvability are jointly increased. This phenomenon was clearly demonstrated by computational studies of structure prediction models and corroborated in network models. Ahnert [173] provides a detailed review of these robustness and evolvability results for these and other GP-mapping models.

Robustness and evolvability play critical roles in the evolution of cancers. Indeed, it is insufficient robustness to mutation that drives oncogenesis, often through two or more mutational hits. Furthermore, as tumours grow they accumulate mutations, both neutral and functional, that render the cancer cell population heterogeneous and primed to evolve resistance once therapy begins. Intuition gained from simpler GP-mapping models can help in understanding this process, and potentially provide evidence for a change in strategy. For example, the RNA model suggests that some phenotypes are accessible through relatively few mutations regardless of the starting genotype. If this is the case for drug-resistant phenotypes, then resistance may be near-inevitable and a therapeutic strategy such as adaptive therapy [8] that focuses on managing resistance may be preferable. Alternatively, since robustness or evolvability are themselves evolving, we may be able to alter the degree of evolvability in a disease by first attempting to steer the evolution through a sequence of drugs.

#### 4.1.3. Epistasis, modularity and pleiotropy

Studies of empirical fitness landscapes have highlighted the prevalence of epistasis and mathematical arguments show that reciprocal sign epistasis can induce rugged fitness landscapes, limiting the number of accessible evolutionary trajectories, and rendering evolution potentially predictable. These results motivate the concept of evolutionary steering [14,174], wherein drug sequences are prescribed to drive the evolution of a disease to a phenotype that is more readily treatable, while avoiding the emergence of highly resistant strains. Higher-order epistasis, wherein the fitness contribution of a given mutation is dependent on the alleles at more than one other loci, is also commonly observed, although the implications of this, especially with respect to the predictability of evolution, have yet to be fully determined [87,175]. In cancer drug discovery, identifying genes that exhibit reciprocal sign epistasis with an oncogene serves as a means to identify potential targets through synthetic lethality. Specifically, inhibiting the action of such a gene can simulate a mutation which, coupled with the already mutated oncogene, is lethal where neither mutation alone is. Extending this approach to account for higher-order epistasis could help identify effective combination therapies if no sufficient pairwise genetic interaction can be identified.

Model GP-mappings also provide insight how modularity and pleiotropy evolve. Fisher’s geometric model suggests that mutations to highly pleiotropic genes are less likely to be beneficial and therefore evolution will proceed more slowly when organismal phenotype is less modular [74]. This phenomenon has been called the ‘cost of complexity’ and there is some empirical evidence to support this hypothesis [176]. However, future studies of this phenomenon will require a much deeper understanding of organismal phenotypes and their dependence on genetics. While molecular reductionism has generated a wealth of genetic (and proteomic, metabolomic etc.) data, the nature of cellular phenotypes is, comparatively, poorly understood. This represents a problem for all studies of evolution, as well as for making evolutionary predictions in cancers and other diseases, as phenotypes are the ultimate determinants of fitness. Recently, Arias *et al.* [177] introduced the model system toyLIFE which comprises a simplified model of chemistry governing gene expression and the interactions between proteins. In this model, the GP-mapping is multilevel with phenotypes arising from a process of transcription, protein folding and protein–protein interaction. Robustness and evolvability have been explored in this model [178] and it captures many of the properties arising in other GP-mapping models. We anticipate that toyLIFE may prove a useful tool for exploring if there are limitations of data gathered from one stage of the GP-mapping in predicting downstream phenotypes where pleiotropy is present.

#### 4.1.4. Bet-hedging

A major assumption underpinning many models of the GP-mapping is that phenotypes result from genotypes in a deterministic way. The appeal of this deterministic assumption is that we can ignore the phenotypes and characterize diseases purely at the more easily quantified genetic or molecular level. However, there is clear evidence that the genes are not the sole determinant of phenotype and that the ‘genes as blueprints’ model is flawed [30]. Bacterial persisters [22] and their recently discovered analogue in cancer [179,180] suggest that some aspects of organismal phenotype may be stochastically determined. This phenomenon, known as bet-hedging, has been demonstrated to have important implications for the progression of disease and the evolution of drug resistance. New models that directly account for the stochasticity in the GP-mapping, and in which the magnitude of this stochasticity is subject to evolution, are needed if we are to understand the evolution of bet-hedging. Further, to manage diseases with drug resistance driven by bet-hedging we will need to design experiments to better identify bet-hedging and simulation studies with model GP-mappings will likely prove a useful tool. Our own previous work in bet-hedging driven by stochastic intra-cellular interaction networks represents a step towards this end [170].

#### 4.1.5. Phenotypic plasticity

Phenotypic differences among genetically identical individuals can also be by driven by environmental influence, endowing a species with robustness to environmental change within certain limits. This phenotypic plasticity has been observed as a driver of drug resistance in both cancers and bacterial infections, for example, through the development of efflux-pumps in response to drug-treated environments [25]. We find that plasticity is poorly represented in GP-mapping models and that the environment is often over-simplified. For example, the environment is often taken as a single target ‘optimal’ phenotype and fitness as some measure of difference from this phenotype. This approach is clearly flawed. Firstly, we know that a single optimal solution to any ‘problem’ rarely exists. Examples include the Spemann organizer discussed earlier [145,146], or the convergent evolution of eyes or wings [181]. Secondly, even where a single globally optimal phenotype exists, there is no guarantee that evolutionary trajectories towards this phenotype will not become trapped at suboptimal solutions owing to rugged fitness landscapes. Finally, we note that this definition of environment is flawed as it plays no role in the determination of phenotypes and, thus, phenotypic plasticity (and development) is ignored. These issues are partially mirrored in our experimental approach to understanding cancer. Tumours are catalogued extensively with respect to their genotype though sequencing and their phenotype through immunohistochemistry, but quantification of the tumour microenvironment still remains challenging.

The neural network model of Gerlee & Anderson [159] partially overcomes this problem by considering a complex environment of diffusible factors that explicitly influence the phenotypes of cells within a growing solid tumour. These factors are spatially heterogeneous and, coupled with constraints on growth driven by crowding effects, are also responsible for driving natural selection and determining the fitness of individual cells. The drawback of this more realistic environmental modelling is that fitness itself cannot explicitly be defined but is rather emergent from simulation of the entire system. This complexity limits the analysis of large numbers of the drug combinations or timing strategies required to design adaptive therapies through the phase *i* paradigm [182]. The future of modelling phenotypic plasticity will lie in balancing complex environmental influence on phenotype with tractable models of fitness and evolution. One current approach to this problem is the development of statistical methods for the analysis of discrete cellular automaton methods [183].

## 5. Conclusion

We have presented a generalized evolutionary framework that provides a lens through which to compare GP-mapping models arising from distinct subfields of computational biology. By considering models under this common framework, we have found that a number of evolutionary predictions are conserved between models, lending credence to their applicability to genuine biology. Furthermore, our survey highlights aspects of the GP-mapping that are unaccounted for, or underrepresented, in theoretical studies. Specifically, the non-functional aspects of the GP-mapping, wherein a single genotype can give rise to a number of phenotypes either stochastically (bet-hedging) or through environmental modulation (plasticity), are presently understudied, despite the observed role of these phenomena in the evolution of drug resistance. The need for models displaying phenotypic plasticity was highlighted by Pigliucci [30] as a necessary step towards reconciling developmental biology with the modern evolutionary synthesis and moving away from the reductionist ‘genes as blueprints’ perspective. Towards this end, a critical first step will be to introduce models of both phenotype and environment that capture sufficient biological complexity while remaining computationally tractable.

Finally, we note that our survey of theoretical modelling clearly demonstrates the power of mathematical models of the GP-mapping in clarifying seemingly paradoxical evolutionary phenomena. For example, the robustness/evolvability trade-off, the evolution of modularity, or the maintenance of bet-hedging as a survival mechanism in fixed environments where it is deleterious [170]. In future, modelling will continue to elucidate unintuitive properties of evolution, particularly in the emerging field of evolutionary medicine. If we are to be able to predict evolution, as we must to design effective therapies for cancer and microbial infections, then characterizing unintuitive properties of evolution will be key in interpreting experimental or clinical observations.

## Endnotes

1 In mathematical terminology a ‘map’ should associate only a single phenotype with each genotype (i.e. be a function). However, for some models inspired by biology this need not be the case (see box 1, non-genetic heterogeneity) and thus strictly speaking the GP-mapping is a relation (or mapping to the powerset of phenotypes). Despite this issue of mathematical terminology, we adhere to the naming ‘GP-mapping’ as it is the established terminology but emphasize that it is, in fact, a relation in the mathematical definition.

2 We note that oscillatory dynamics of protein expression are in fact common, for example, in the cell cycle [147], and thus it is not clear whether this definition of phenotype is too restrictive.

## Data accessibility

This article does not contain any additional data.
